# A Giant Childhood Mesenteric Lipoblastoma With Extensive Maturation

**DOI:** 10.3389/fped.2020.00404

**Published:** 2020-07-24

**Authors:** Anthony I. Squillaro, Monica D. Chow, Fernando Arias, Evita T. Sadimin, Yi-Horng Lee

**Affiliations:** ^1^Department of Surgery, Rutgers Robert Wood Johnson Medical School, New Brunswick, NJ, United States; ^2^Department of Pathology, Rutgers Robert Wood Johnson Medical School, New Brunswick, NJ, United States; ^3^Division of Pediatric Surgery, Rutgers Robert Wood Johnson Medical School, New Brunswick, NJ, United States

**Keywords:** soft tissue tumors, lipoblastoma, lipoma, mesenteric tumors, pediatric tumors

## Abstract

Abdominal lipoblastomas are uncommon soft tissue tumors in children and rarely arise from the mesentery. Due to intraabdominal location and slow growth, these masses can go unnoticed for long periods of time and often found on surgical exploration. We present a case of a 12-year-old male with years of abdominal distension accompanied by new onset early satiety that was found to have an intra-abdominal mass. He underwent an exploratory laparotomy revealing a large 33 x 27 x 15 cm rubbery mesenteric mass displacing the entire intra-abdominal contents, connected by a single vascular pedicle and encasing a loop of small intestine. The mass was resected and the patient did well without signs of recurrence. Histology confirmed the presence of mature adipocytes but on further cytogenetic analysis, a translocation between chromosomes 2 and 8 at the 12q arm was detected, which is often associated with lipoblastomas. This case represents the one of the largest mesenteric lipoblastomas that matured extensively to lipoma-like histology at the time of surgical resection.

## Introduction

Lipoblastomas are an uncommon soft tissue tumor of infancy and early childhood. These tumors are composed of fetal lipoblasts that continue to proliferate in the post-natal period and can clinically mimic the more common lipomas seen in older patients (1, 2). Most cases are found in infants and young children, and only rarely are they seen in adolescents (3, 4). While fetal lipoblasts are commonly seen on histology, they can be morphologically classified into several subtypes according to histological differences. The classic lipoblastoma has been described to contain primitive mesenchymal cells, while those that undergo extensive maturation are lipoma-like. They lack a myxoid component and are comprised predominantly of mature adipocytes (5). We report a 12-year-old male who had a long-standing history of abdominal distension and presented to our institution with new onset early satiety for several weeks. An exploratory laparotomy revealed a giant rubbery mesenteric mass was resected. His histology confirmed the presence of mature adipocytes consistent with lipoma, but had a karyotype characteristic of a lipoblastoma, suggesting that this lipoblastoma underwent extensive maturation by age of diagnosis and subsequent resection.

## Case Report

A 12-year-old male with a history of a distended abdomen presented to the emergency department (ED) after outpatient imaging studies were significant for an abdominal mass. He initially presented to his gastroenterologist with a 2-week history of intermittent epigastric pain that, worsened after each meal. He also developed early satiety and his parents reported increased distention. Review of systems was otherwise negative. Of note, the patient had a protuberant abdomen starting at 3 years of age. Two ultrasounds were completed at that time and both were reportedly negative for any findings; the images were not available for review. Over the years, his abdomen gradually became more distended.

**Figure 1:**
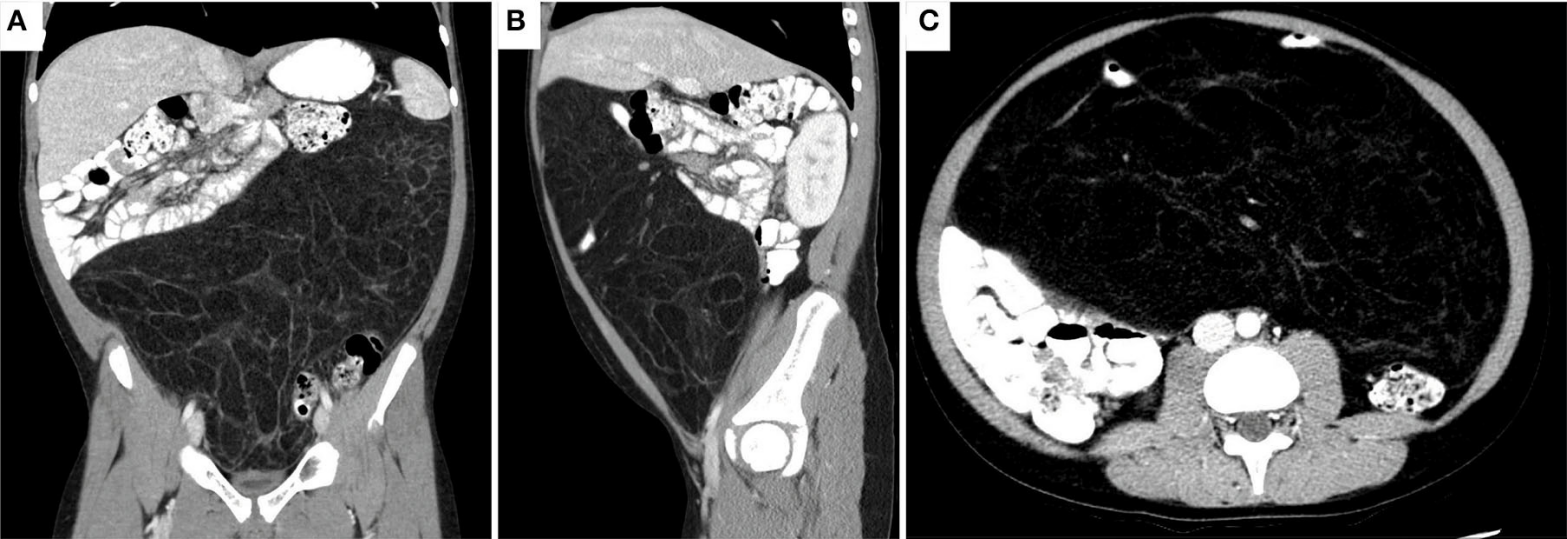
CT abdomen/pelvis revealing a 33 x 27 x 15 cm, predominant fatty mass with multiple septa and a few areas of soft tissue density. The mass displaces most of the abdominal contents posteriorly. A loop of small bowel is seen within thin the mass. No evidence of obstruction noted as oral contrast reached the sigmoid colon. **(A)** Coronal **(B)** Sagittal **(C)** Axial views.

He underwent an abdominal X-ray, which was significant for an abdominal mass. He was then sent for a CT of the abdomen and pelvis, which again noted an abdominal mass 27 x 33 x 15 cm in size that displaced the majority of his abdominal contents posteriorly and that appeared to be mesenteric in origin (Figure 1). He was instructed to go to the ED after the CT findings. On physical exam, his vital signs were within normal limits and his abdomen was soft, non-tender to palpation, and distended. There were no palpable masses in the abdomen or palpable lymph nodes. His blood work was also unremarkable.

**Figure 2:**
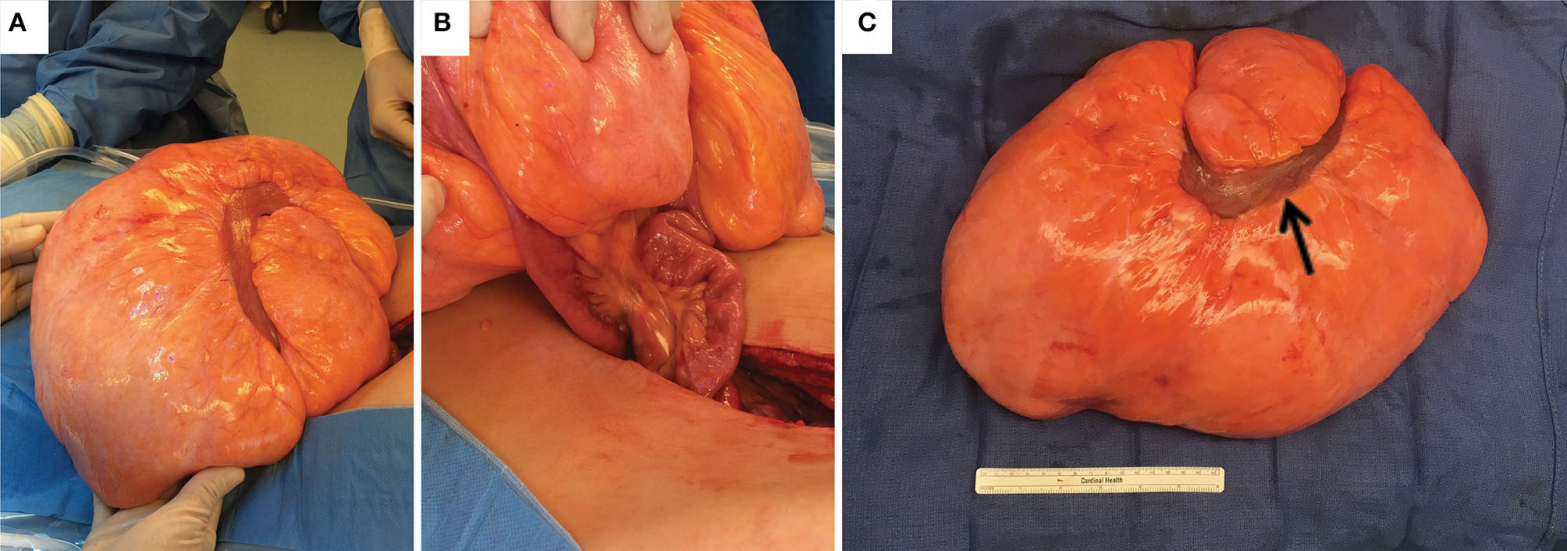
Images of the gross specimen. **(A)** mesenteric lipoma *in situ*. **(B)** The lipoma was connected to a single pedicle of mesentery accompanied by a loop of small bowel entering through the mass. **(C)** Gross specimen after resection with one loop of small bowel (black arrow).

Hematology/oncology was consulted, and a CT neck and chest were obtained to look for potential metastatic disease, both of which were negative. The patient was admitted and underwent surgical resection of the mass the following day. On exploration, the mass was tan-yellow in color, firm with a rubbery consistency, involved a segment of small bowel, and was connected via a single vascular pedicle (Figure 2). The vascular pedicle was ligated, and the involved segment of small bowel was resected along with the entire mass. Abdominal exploration revealed no other abnormalities. The patient did well postoperatively, resuming a liquid then regular diet, had return of bowel function, and was discharged. The patient was seen in the clinic shortly after, was doing well, and had total resolution of his symptoms.

Pathological examination revealed that the mass arose in the mesentery of the small bowel, measuring 38 x 23 x 12 cm and weighing 5 kg. On thorough sectioning, the mass was well-circumscribed and composed entirely of lobulated adipose tissue without any fibrous septa. The small bowel was 30 cm in length with unremarkable mucosal and serosal surfaces.

**Figure 3:**
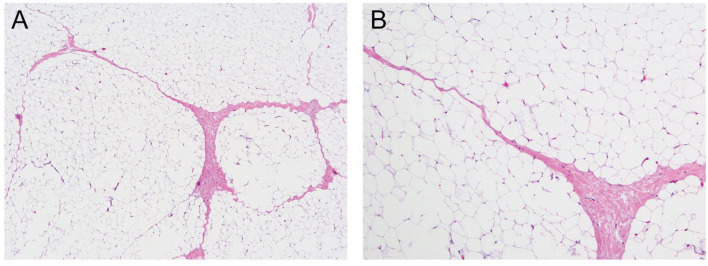
Mature lobulated adipose tissue, hematoxylin and eosin stain under 40X magnification **(A)** and 200X magnification **(B)**.

Morphologically, the lesion was composed entirely of mature lobulated adipose tissue, with delicate fibrous bands surrounding the lobules, without any immature component (Figure 3). The lesion did not show any atypia, necrosis, or mitosis. Karyotyping revealed translocation between chromosomes 2 and 8 in all cells [46, XY, t (2,8)(q12; q12)]. More specifically, the rearrangement involved q12 of chromosome 8, the region of *PLAG1*, which is the characteristic abnormality in lipoblastoma. This finding is consistent with lipoblastoma with complete maturation, thereby resembling a lipoma on histology.

## Discussion

Adipose tissue tumors are uncommon in the first two decades of life, with lipoma as the most common mesenchymal tumor overall. Lipoblastomas are comprised of immature adipocytes, rare, mostly benign, and over 80% present before 3 years of age (1, 6). They are most commonly located in the extremities. Abdominal lipoblastomas represent only 7% of all lipoblastomas, with the majority of them arising from the retroperitoneum (7–9). The description of lipoblastomas maturing into lipomas was first described by Van Muers (10). Over a 2-year period, he performed 5 partial excisions of an axillary lipoblastoma and on final histology, these lesions showed histological transformation into mature lipoma. Like mesenteric lipoblastomas, a mesenteric lipoma is exceedingly rare with only several dozen cases described in the literature (11–13). Symptoms are uncommon and variable, most often owing to enlargement. They can include distension, abdominal pain, vomiting, ileus, and even intestinal obstruction due to volvulus (14). In a review of approximately a dozen cases of mesenteric lipomas presenting with abdominal pain, complete intestinal obstruction secondary to mass compression was found in lipomas with mean size of 21 cm, while obstruction caused by volvulus was found in smaller lipomas with a mean size of 13 cm (11). Due to the location and slow growth of mesenteric lipomatous tumors, these masses often go unnoticed for long periods of time. Long standing abdominal protuberance with new onset of early satiety, as in our patient, was attributed to the space occupying and compressive nature of the lesion. To our knowledge, this is the largest mesenteric lipoblastoma with extensive adipocyte maturation resembling a mature lipoma documented in the literature to date. Even the largest documented mesenteric lipoma in the pediatric population to date is 28 cm in the largest dimension (15).

O'Donnell et al. proposed renaming lipoblastomas as “infantile lipoma” since the term “blastoma” is generally reserved for tumors that metastasize (16). In their review of the literature, they found that these tumors have no reports of metastasis and have an ability to mature into simple lipomas as their cytoarchitecture can eventually be replaced by mature adipocytes. While the presence of lipoblasts aids in diagnosis, lipoblastomas can feature a wide range of adipocyte differentiation, in which vacuolated lipoblasts can be mixed in with mature adipocytes. The variety of lipoblasts and mature adipocytes found in these tumors has led to a theory that lipoblastomas can transform into mature lipomas. The current case reflects that phenomena, as maturation likely occurred due to presentation at adolescent age. Thus, the term “infantile lipoma” could be confusing, as the patient presented later than typical lipoblastomas of early childhood. Additionally, since lipoblastomas have an increased risk of recurrence as compared to lipomas, the retention of “lipoblastoma” nomenclature has been advocated to clearly differentiate these lesions from typical lipomas (2). The continued importance of this classification should prompt clinicians of the necessity to completely excise these lesions and follow-up with patients for required monitoring for lipoblastoma recurrence.

A distinctive feature of this case is not only the giant size of the tumor, but that cytogenetics uniquely identified this tumor's pathogenesis extensive maturation. Lipomatous tumors can have normal karyotypes; however, there have been a number of tumor specific chromosomal translocations and associated fusion genes that have been identified in adipocyte tumors. For example, there exists chromosomal rearrangements at regions that include 12q14–15, 6p21–23, 13q2–22 (17, 18). Re-arrangement of chromosome 8 at band q12 is a characteristic cytogenetic abnormality found in lipoblastomas, and the gene at the breakpoint region is *PLAG1* oncogene, which is prevalent in 70% of lipoblastoma tumors (5). *PLAG1* encodes a zinc finger transcription factor expressed primarily in fetal tissue and at low levels postnatally (19). Through promoter swapping, *PLAG1* becomes transcriptionally upregulated and frequently partnered to genes hyaluronic acid synthase 2 *(HAS2*) on chromosome 8 and collagen 1 alpha 2 (*COL1A2)* on chromosome 7, with additional genes possible as well (20). While translocations have been reported between chromosome 2:8 in at least one gluteal and mesenteric lipoblastoma, they occurred at different breakpoints t (2,8) (q23; q24) and t (2,8) (q23; q11.2), respectively (17, 21). The resulting fusion gene from chromosomal translocation in this case is thus unknown but may result in chimeric genes causing transcriptional deregulation as has been implicated in other soft tissue tumors (22, 23).

Tumor-defining genetic abnormalities such as translocations and the resultant genes affected can be particularly beneficial in cases where tumor histopathology may appear similar, but genetic tumor identity carries vastly different treatment implications. For the adolescent patient group with a large lipomatous tumor like the one described here, this distinction may be crucially important. Perhaps a critical example is in distinguishing between a benign lipoma and a malignant well-differentiated liposarcoma as the incidence of these malignant lipomatous tumors become more common in the second decade of life and both contain mature adipose cells. While both these tumors share a cytogenetic abnormality at 12q13-15 chromosome region, they carry vastly different prognoses, as a well-differentiated liposarcoma as malignant and metastatic potential (2). Knowledge of these cytogenetic and genetic signatures can be crucial in truly determining tumor identify in cases where histopathology is similar, but the treatment and follow-up for patients is divergent.

## Conclusion

This case represents the one of the largest known mesenteric lipoblastoma with extensive maturation on histology to be resected in a child. While mature adipocytes were present on histology, cytogenetic analysis revealed a novel karyotype that more closely resembled a lipoblastoma. Additional studies may help further understanding of the role of karyotypes in distinguishing between lipomas and lipoblastomas that undergo extensive maturation by the age of clinical resection.

## Data Availability Statement

The original contributions presented in the study are included in the article, further inquiries can be directed to the corresponding author/s.

## Ethics Statement

Written informed consent was obtained from the minor's legal guardian for the publication of any potentially identifiable images or data included in this article.

## Author Contributions

AS, Y-HL, and ES participated in the care of the patient. AS and MC collected, analyzed, and interpreted the data and drafted the manuscript. FA collected and interpreted the data and critically revised the manuscript for important intellectual content. ES and Y-HL interpreted the data and critically revised the manuscript for important intellectual content. Y-HL was the lead physician in the care of this patient and supported the study. All authors approved the final manuscript as submitted and agree to be accountable for all aspects of the work.

## Conflict of Interest

The authors declare that the research was conducted in the absence of any commercial or financial relationships that could be construed as a potential conflict of interest.
